# Development of Methods for the Synthesis of Neopentyl Glycol by Hydrogenation of Hydroxypivaldehyde

**DOI:** 10.3390/molecules26195822

**Published:** 2021-09-25

**Authors:** Edyta Monasterska, Anna Chrobok, Ewa Pankalla, Agnieszka Siewniak

**Affiliations:** 1 Department of Chemical Organic Technology and Petrochemistry, Ph.D. School, Silesian University of Technology, Akademicka 2A, 44-100 Gliwice, Poland; edyta.monasterska@polsl.pl or; 2 Grupa Azoty Zakłady Azotowe Kędzierzyn, S.A., Mostowa 30A, 47-220 Kędzierzyn-Koźle, Poland; ewa.pankalla@grupaazoty.com; 3 Department of Chemical Organic Technology and Petrochemistry, Silesian University of Technology, Krzywoustego 4, 44-100 Gliwice, Poland; anna.chrobok@polsl.pl

**Keywords:** neopentyl glycol, hydroxypivaldehyde, hydrogenation, crossed aldol condensation

## Abstract

Neopentyl glycol (NPG) is a precursor for the manufacture of many valuable products of industrial importance such as polyester, polyurethane and alkyd resins, synthetic lubricants, hydraulic fluids, drugs, etc. The structure of NPG provides the resins with excellent hydrolytic stability, resistance to weather conditions, good flexibility-hardness balance, and outstanding functional properties. The paper presents a literature review on the development of methods for NPG preparation, focusing primarily on the synthesis of NPG by hydrogenation of hydroxypivaldehyde, which is obtained by the crossed aldol condensation of isobutyraldehyde and formaldehyde. Preparation of the substrates, catalysts, technical and apparatus solutions, and NPG purification were discussed.

## 1. Introduction

**Figure 1:**
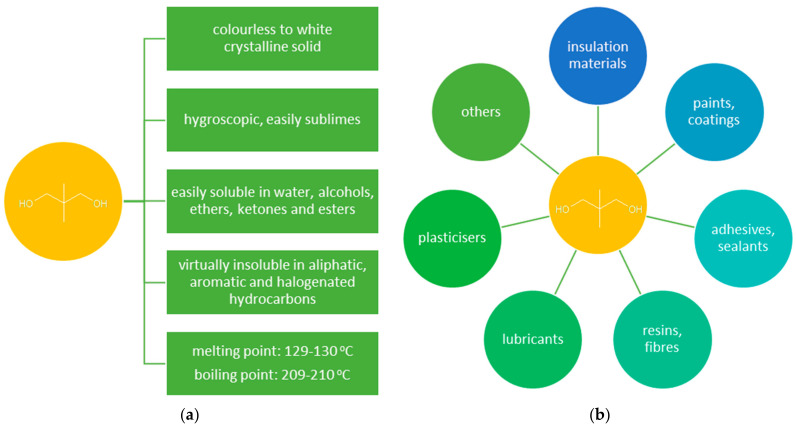
(**a**) Properties of NPG [2]; (**b**) applications of NPG.

The unabated growth in demand for high-quality polymeric materials used in various industries resulted in a growing interest in neopentyl glycol (NPG, 2,2-dimethylpropane-1,3-diol) (Figure 1a). NPG is widely used for the preparation of saturated and unsaturated polyester resins, polyethers, polyurethanes, and high-quality alkyd resins [1,2]. NPG is also used for the production of synthetic lubricants, drugs, pesticides, plasticizers, paints, varnishes, and dyes (Figure 1b) [3,4,5]. The introduction of a neopentyl structure to the NPG-derived products enhances their stability towards hydrolysis, high temperature, and light exposure [3]. Therefore, NPG finds application, in particular, in the synthesis of high-quality coatings (mainly solvent-free powder coatings) that are used, e.g., in the automotive, construction, and shipbuilding industries [6,7,8,9]. Moreover, NPG shows properties characteristic of phase changing materials (PCMs) (solid-solid phase transition at 43 °C) [9,10]. PCMs are used in many fields due to their ability to accumulate and release energy in the temperature range of the phase transition.

The key NPG producers are BASF SE, Eastman Chemical Company, Mitsubishi Gas Chemical Company, Perstorp Group, LG Chem Company, Inc, Oxea GmbH, Celanese Corporation, Shandong Dongchen, etc. [11]. In 2018, the NPG market was estimated at USD 3.25 billion [11] and is expected to augment in the near future. The global production capacity was about 220,000 t/a in the year 2000 [2] and 400,000 t/a in the year 2008 [12].

In the absence in recent years of reviews concerning the NPG synthesis, this paper focuses on the development of industrial methods of NPG preparation by the crossed aldol condensation of isobutyraldehyde (IBA) and formaldehyde (FA), as well as the subsequent hydrogenation of the obtained hydroxypivalaldehyde (HPA, 2,2-dimethyl-3-hydroxypropanal).

## 2. Synthesis of NPG

**Figure 2:**
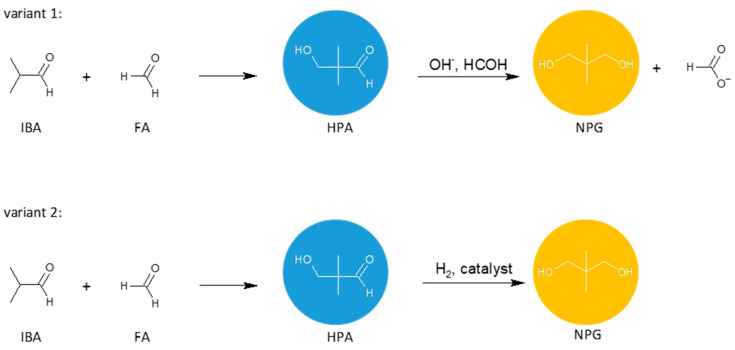
General schemes of NPG synthesis: condensation of isobutyraldehyde with formaldehyde and the subsequent Cannizzaro reaction (variant 1) or hydrogenation of HPA (variant 2).

Neopentyl glycol can be obtained by the crossed aldol condensation of isobutyraldehyde with formaldehyde or paraformaldehyde to produce hydroxypivaldehyde as an intermediate. Then, HPA can be converted to NPG either by a Cannizzaro reaction with formaldehyde (Figure 2, variant 1) or by a hydrogenation reaction (Figure 2, variant 2). These two methods are of industrial importance. Other methods of NPG synthesis are: dimethylmalonic acid or esters reduction with hydrogen or lithium aluminum hydride [13], 2-methyl-1,2-epoxypropane hydroformylation, and the subsequent reduction [14].

In variant 1, the condensation reaction and the Cannizzaro reaction can be carried out in one or two separate stages. The Cannizzaro reaction, which is performed in the presence of a concentrated inorganic hydroxide solution, gives apart from NPG, an equimolar amount of formic acid which is neutralized by the alkaline catalyst. This salt has no useful application, making the method ineffective and industrially unattractive. Recently, however, an interesting method for carrying out polyol synthesis via the Cannizzaro reaction was disclosed in the patent by Oxea [15]. In this method, two different polyols are obtained simultaneously in the reaction of two different aliphatic aldehydes with formaldehyde in the presence of aqueous solutions of KOH, NaOH or Ca(OH)_2_. The process takes place in two stages. In the first stage, FA reacts with the first aldehyde, using a 3- to 12-fold excess of formaldehyde relative to the aldehyde, and the reaction is carried out at a temperature ranging from 20 to 65 °C until at least 50% conversion of aldehyde is achieved. Then, without any work-up and isolation, the reaction mixture is reacted with the second aldehyde at a temperature ranging from 30 to 75 °C, with a molar ratio of FA to aldehyde being 2:1 to 7:1. The process is carried out until the aldehydes are fully converted. According to the authors, the simultaneous consecutive synthesis allows the attainment of polyols with high yields and selectivity.

In variant 2, the first step—condensation of isobutyraldehyde with formaldehyde is commonly conducted in the presence of a basic catalyst such as trialkylamines [16,17,18,19,20], sodium or potassium carbonates [21], anion exchangers [3,22] or layered double hydroxides [23,24]. The selectivity of HPA synthesis using tertiary amines as catalysts is usually higher compared to the processes carried out with inorganic bases [16]. However, as with inorganic bases, tertiary amines can form salts by the reaction with organic acids. These acids are mainly formed in the Cannizzaro reaction or are introduced with starting materials, e.g., formic acid from commercial formaldehyde which is usually used as an aqueous solution containing methanol. The resulting salts cannot be easily separated from the hydroxyaldehyde and are usually directed with hydroxyaldehyde to the hydrogenation reactor. Their presence in the HPA stream may lead to deactivation of the metal catalyst which is used in the hydrogenation reaction. Moreover, they may promote the decomposition of the aldol condensation product during the distillation of the reaction mixture at high temperatures, thereby reducing the yield of the desired product. Amine salts can also adversely affect the color and/or odor of HPA derived products [16]. 

**Figure 3:**
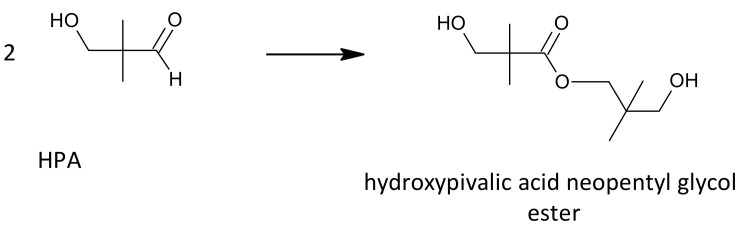
Tishchenko reaction on the example of hydroxypivalaldehyde as a substrate.

Another competing side reaction that can occur during the aldol condensation is a Tishchenko reaction (Figure 3), in which an ester is formed from two aldehyde equivalents as a result of aldehyde disproportionation, e.g., hydroxypivalic acid neopentyl glycol ester (hydroxyneopentyl hydroxypivalate). 

**Figure 4:**
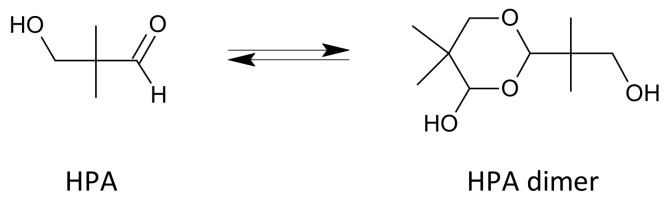
Hydroxypivalaldehyde and its dimer.

The aldol condensation is usually carried out in the presence of an excess of one of the aldehydes, which is often distilled off after the reaction with the trialkylamine and then recycled to the process. An additional solvent is often used, e.g., an aliphatic alcohol, and the reaction is typically carried out at temperatures ranging from 20 to 100 °C [25]. Crude HPA is sometimes purified by distillation and/or extraction prior to being sent to the hydrogenation reactor. Hydroxypivaldehyde easily dimerizes (Figure 4) [18,26]. At higher temperatures, the equilibrium is shifted towards the monomer and, after cooling, towards the dimer [26]. The dimer is a solid with a melting point of 89–90.5 °C [26].

The second step involves the hydrogenation of HPA. Typical reaction conditions range from 50 to 250 °C and from 0.1 to 30 MPa. The process may be carried out in the presence of a solvent or solvent mixture. Metal catalysts are mainly used for the hydrogenation step, e.g., Cu, Ni, and Ru. Product purification is usually performed by distillation, extraction or crystallization.

This work focuses on variant 2 involving the synthesis of neopentyl glycol by the hydrogenation of hydroxypivaldehyde, which in turn is obtained from formaldehyde and isobutyraldehyde. Although the chemical basics of NPG preparation are known, the main challenges to be addressed in developing a method for NPG synthesis according to variant 2 include:

- Improvement of the aldol condensation step by developing an active, selective, and easy to separate catalytic system;
- Improvement of the intermediate HPA separation step—the impurities may deactivate the hydrogenation catalyst and promote the degradation of HPA;
- Development of a selective, life span and easy to remove hydrogenation catalyst;
- Improvement of NPG purification methods—high purity of NPG is required in the synthesis of many products derived from NPG;
- Apparatus improvements.

Examples of attempts to solve the above-mentioned problems are presented below.

### 2.1. Improvement of the Production and Purification of HPA

**Figure 5:**
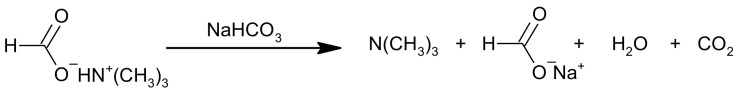
Scheme of reaction between NaHCO_3_ and trimethylamonium formate salt [16].

In order to improve the preparation of polyols, including NPG, the Eastman company has developed a method that allows the reduction of the amount of salts formed from carboxylic acids and tertiary amine at the stage of HPA synthesis [16]. The presence of these salts in the hydrogenation step may contribute to the decomposition of a metal catalyst. To avoid this, the addition of a basic promoter to the aldol condensation reactor has been proposed. The role of the promoter is to enhance the dissociation of nitrogen-containing salts and increase FA conversions (Figure 5). Therefore, it must show an appropriate pKa value. The promoter also reduces the consumption of the trialkylamine catalyst and improves its recovery. Among others, carbonates, bicarbonates, hydroxides of alkali metals, and alkaline earth metals, i.e., Li_2_CO_3_, Na_2_CO_3_, CaCO_3_, LiHCO_3_, KHCO_3_, NaOH, KOH, Ca(OH)_2_ can be used as promoters. The promoter can be used as an aqueous solution with a concentration ranging from 5% to about 50%. The amount of promoter should be sufficient to lower the total nitrogen content of the stream directed to the hydrogenation to 25 ppm or less. The crude HPA may be additionally purified by distillation before being directed into the hydrogenation reactor, in order to remove the low-boiling reagents and catalyst. The second step—the hydrogenation process can be carried out in the presence of catalysts such as NiMo, NiCo, CuCr, CoMo, and other metal catalysts. 

The patent [27] describes a method for the preparation of NPG, in which the aldol condensation step is carried out in the presence of weakly basic anion exchange resins with -NH_2_, -NHR or -NR_2_ groups, where R represent the alkyl or aryl groups. The reaction proceeds at a temperature of 15–100 °C using a molar ratio of IBA to FA of 15:1–1:15. This step may be performed in the presence of a solvent such as alcohols or ketones. The obtained HPA without any purification is hydrogenated in the presence of the catalyst, e.g., copper chromite, oxides of cobalt, manganese, nickel, Pt, Ru, W, and Pd. The reaction is carried out in a solvent, e.g., alcohol, ether, ketone, in an amount of 1 to 70 wt%, at a temperature of 50 to 200 °C and pressure of 0.1 to 20 MPa. The product is then separated from the reaction mixture by conventional methods, e.g., distillation. 

The patent by ClearWaterBay Technology describes a method of NPG synthesis in which a solid basic catalyst, e.g., an ion exchange resin, zeolite, is used in the aldol condensation stage [3]. Due to the limited solubility of isobutyraldehyde in the aqueous FA solution, the starting materials should be introduced into the aldol reactor as a homogeneous solution. This can be achieved by maintaining the appropriate ratio of IMA: FA and water or by adding a solvent, e.g., methanol, ethanol, isopropanol, ethylene glycol or NPG, in which both isobutyraldehyde as well as FA and water would dissolve. The addition of NPG is preferred since this avoids the addition of further solvents to the mixture. NPG is introduced in the amount of 10–30 wt%. Using Amberlyst A21 as a catalyst, the high FA conversion (88.9%) and selectivity (94.9%) were achieved. The process is carried out at the temperature of 80 °C under atmospheric pressure. The crude product is distilled to remove the unreacted starting materials which are recycled to the reactor. The second stage—HPA hydrogenation is carried out in the presence of copper chromite or ruthenium on activated carbon (containing Cu, Co, and Mn). The hydrogenation process is conducted at a temperature in the range from 110 to 170 °C and pressure of 2 MPa. The molar ratio hydrogen to hydroxypivaldehyde can be in the range from 10:1 to 3:1. The hydrogenation reaction takes place in a stainless steel tubular reactor 300 mm in length and 10 mm in diameter filled with the catalyst. Conversion of hydroxypivaldehyde was 98.1% with 99.3% selectivity to NPG. To purify the NPG, either crystallization or distillation is performed.

The patent by LG Chemical disclosed a method that allows the attainment of neopentyl glycol with a purity ≥98%, which consists of removing the unreacted raw materials, catalyst residues (trialkylamine), and by-products after the aldol condensation step [20]. This is achieved by extracting the HPA condensation product with an aliphatic alcohol, preferably octanol, and then the resulting extract is distilled to remove compounds with a boiling point lower than that of HPA. Only this purified HPA is directed to the hydrogenation reactor. This step is carried out in the presence of a nickel catalyst, e.g., Raney nickel, in a reactor, which allows the recycling of part of the hydrogenation product, at a temperature of 120 to 180 °C and under pressure of 0.7 to 10.3 MPa. After hydrogenation, the mixture can be saponificated to convert neopentyl esters to NPG. Then, the post-reaction mixture is extracted with water. The extract, which mainly contains neopentyl glycol and water, in a weight ratio of 4:6 to 9:1, is subjected to azeotropic distillation. 

BASF has disclosed in its patent a method for the continuous liquid phase hydrogenation of HPA, in which the hydrogenation reactor feed is a mixture consisting of: An aldol condensation reactor stream containing crude HPA, a recycle stream from the hydrogenation reactor, and a pH regulator stream used to maintain the pH from 7 to 9 at the outlet of the hydrogenation reactor [6]. Providing an appropriate pH in the hydrogenation reactor allows the reduction of side reactions, e.g., Tishchenko reaction, hydrolysis, formation of acetals, etc. and extends the life of the hydrogenation catalyst. For example, formic acid, which is present in the HPA stream due to the Cannizzaro reaction, decomposes into CO_2_ and H_2_ or CO and H_2_O during the hydrogenation. However, as the activity of the hydrogenation catalyst decreases, the rate of the acid decomposition reaction also decreases and, as a result, the pH of the reaction mixture changes. Therefore, in the patent, the use of the additive compound to control the pH is proposed, which may be, depending on the needs, an inorganic or organic base, e.g., trialkylamine, Na_2_CO_3_, K_2_CO_3_, CaCO_3_, NaHCO_3_ or inorganic or organic acids such as sulfuric acid or phosphoric acid, citric acid, and acetic acid. This additive is introduced into the feed stream to the hydrogenation reactor. The amount of pH regulator is determined by the regular analyses of the pH of the reaction mixture. The hydrogenation process is carried out in a continuous reactor in the presence of metals from transition groups, such as Fe, Ru, Os, Co, Rh, Ir, Ni, Pd, Pt, Cu, and most often Ni and Cu, immobilized on a support, e.g., titanium oxides.

Maintaining the proper temperature of the feed to the hydrogenation reactor is important. The too low temperature of the HPA stream causes precipitation of HPA as a solid, thus hindering the flow of the feed. On the other hand, the too high temperature accelerates side reactions (especially Tishchenko reaction) which lower the selectivity and efficiency of the process and shorten the life of the catalyst. In the patent by LG Chem [28], combining the hot effluent from the hydrogenation reactor with the crude HPA stream is proposed in such a way as to maintain a suitable HPA feed temperature. The composition of the HPA feed should be as follows: 6–30 wt% of HPA, 35–70 wt% of NPG, 10–30% of alcohol, 10–30 wt% of water. The hydrogenation can be conducted at a temperature ranging from 100 to 250 °C and under pressure of 10 to 25 MPa. The process is catalyzed by a copper-based catalyst, e.g., CuO (60–99 wt%)/BaO (1–40 wt%), which can be bound to a carrier, e.g., silicon oxide and aluminum oxide. High HPA conversions (99.2–99.8%) and high NPG yield (99.2–99.7%) can be achieved. 

### 2.2. Improvement of the Production and Purification of NPG

The improvement of catalysts is one of the key tools in the development of chemical processes. An increase in the catalyst activity may allow the use of a dilute raw material, and may also improve the conversion of substrates, while an increase in the catalyst selectivity reduces the amount of by-products, which in turn simplifies the separation of the reaction mixture. The catalyst should also be stable under the reaction conditions, resistant to impurities in the feed, and easily separable after the reaction. The development of the hydrogenation catalysts is shown below on selected examples.

One type of catalysts used in the hydrogenation reaction are those based on copper chromite [29,30,31]. Currently, however, such catalysts are losing importance for environmental reasons mainly due to the toxicity of chromium. The Eastman Company developed a low pressure method for the hydrogenation of NPG using a manganese oxide-promoted copper chromite catalyst (CuO:CuCr_2_O_4_) [30]. The low pressure of hydrogen provides a good NPG yield (90.6%). Moreover, the crude hydroxypivaldehyde with by-products from the aldol condensation of isobutyraldehyde with formaldehyde can be directly used for the hydrogenation reactor. There is no need to separate the by-products. However, after the condensation step, the obtained crude HPA product can be separated from the unreacted isobutyraldehyde and triethylamine in the distillation column. The recovered reagents are recycled to the condensation reactor. The hydrogenation step is carried out in two reactors. The feed to the first hydrogenation reactor is crude HPA from the condensation stage combined with the recycled hydrogenated product at a volume ratio of 9:1. The reactors operate at a temperature of 150 to 220 °C and under H_2_ pressure of 2.1 to 10.3 MPa. The hydrogenation catalyst can have the following composition: 45–47 wt% CuO, 45–47 wt% CuCr_2_O_4_, and 2–8 wt% MnO_2_.

Similarly in the Oxea patent, a copper chromite catalyst has been used as the hydrogenation catalyst [31]. In this case, the catalyst is doped with manganese (preferably 3–5 wt%) and barium (preferably 1–4 wt%). Moreover, the 99.9% HPA conversion and 100% selectivity towards NPG can be achieved in a continuous tubular reactor at a temperature of 120 to 135 °C and under hydrogen pressure of 8 MPa. The process is conducted in the presence of the catalyst with the following composition: 47.5% copper, 46.5% chromium, 4.0% manganese, and 2.0% barium, which was mixed with 3% graphite and tableted. The feed to the hydrogenation reactor is crude HPA diluted with an aliphatic alcohol, preferably isobutanol. The solvent is used to ensure the homogenization of the mixture and to prevent the precipitation of HPA during the hydrogenation.

Mitsubishi discloses an active and a long service life catalyst for the synthesis of NPG from HPA containing copper, zinc, and zirconium [32]. The catalyst has an atomic ratio of Zn to Cu and also Zr to Cu of 0.05:1 to 2:1. The hydrogenation process is carried out at 60–250 °C and 0.1–14.7 MPa and in the presence of a solvent which may be an alcohol, an ether or a saturated hydrocarbon. The feed to the hydrogenation reactor can be crude HPA from the condensation step or purified from FA, IBA, and the tertiary amine catalyst by distillation or crystallization. The concentration of hydroxypivaldehyde should be in the range from 15 to 60 wt%. A lower and higher concentration is undesirable. The lower content of HPA in the stream makes the separation of the post-reaction mixture difficult, while the higher content intensifies the Tishchenko reaction. The temperature of hydrogenation reaction ranges from 80 to 200 °C and the reaction pressure from 0.1 to 14.7 MPa. The last step of the process is the separation of neopentyl glycol by distillation or solvent extraction. The process runs with high selectivity (97.6–99.0) and HPA conversion (99.7–100). 

In another method, a catalyst system consisting of copper oxide and zinc oxide with aluminum as a promoter was used for the hydrogenation of hydroxypivaldehyde to neopentyl glycol [33]. This process allows the hydrogenation to be carried out at a temperature of 110 to 180 °C and a moderate pressure of 0.1 to 3.4 MPa, providing 100% aldehyde conversion with 100% selectivity of the desired alcohol. Other examples of the use of copper-based HPA hydrogenation catalysts can be found in the literature [34,35,36,37,38,39,40,41].

Nickel-based catalysts are usually less active at temperatures below 100 °C, while at higher temperatures the formation of larger amounts of by-products is observed. This reduces the selectivity of the process, and the resulting by-products are difficult to remove from the post-reaction mixture. In turn, the lower reaction temperatures require the use of larger amounts of catalyst and longer reaction times, which adversely affects the economic balance of the process. The patent [4] discloses a method for the hydrogenation process using a nickel-containing catalyst but which, however, allows the process to be carried out at a temperature below 100 °C. The nickel content in the catalyst can range from 60 to 99 wt%. In addition to nickel, the catalyst may contain chromium (1–40 wt%) and the catalyst can be applied to the support, e.g., SiO_2_. The process is conducted in the presence of solvents (10–50 wt%), which can be alcohols or ethers or their mixtures. Solvents enable the reaction to be carried out in the liquid phase since they increase the solubility of hydroxypivaldehyde. Water is undesirable in that method as it increases the amount of by-products. Therefore, its content should not exceed 15 wt%. The hydrogenation process can be carried out at 70 °C and a pressure in the range from 1 to 8 MPa, with a good yield of NPG and at least 98% selectivity.

The use of a nickel-based HPA hydrogenation catalyst is also described in the patent [42]. However, in the proposed method, the hydrogenation process can be carried out in the presence of water in greater amounts than 15 and up to 25 wt% based on the overall starting mixture. The use of appropriately high temperature (110 to 180 °C) and pressure (6 to 18 MPa) causes larger amounts of water to contribute to the decomposition of high-boiling by-products, such as esters or cyclic acetals, to NPG, which in turn improves the efficiency and selectivity of the process. The hydrogenation is carried out continuously in a tubular reactor with a fixed catalyst bed. A diluent, most often an aliphatic alcohol, i.e., isobutanol, in an amount of 15 to 27 wt% is added to the crude aldol condensation product to ensure the homogeneity of the input stream.

Scientists from Oxea GmbH have developed a highly active and selective hydrogenation catalyst based on Raney cobalt [43]. This catalyst can be easily separated from the post-reaction mixture and is remarkably stable under the reaction conditions. In addition to cobalt, the catalyst may contain other metals such as: Fe, Ni, Cr, Rh, Ru, Os, Ir, Pt, and Pd. The stream from the aldolization reactor without any treatment purification may be directed to the hydrogenation reactor. Hydrogenation takes place continuously in the liquid phase in the presence of a linear or branched alcohol at a temperature of 60 to 220 °C and a pressure of 2 to 150 MPa. The preferred temperature range is from 70 to 160 °C and a pressure from 10 to 100 MPa.

Noble metals such as Ru and Pt are also used in the hydrogenation of HPA. Ru-supported catalysts on different carriers (Al_2_O_3_, TiO_2_, and activated carbon) were used for the hydrogenation of hydroxypivaldehyde [22]. For each catalyst, the selectivity towards neopentyl glycol was over 99%, but the highest activity showed the catalyst Ru/Al_2_O_3_. The process was carried out at 120 °C under a 5.44 MPa hydrogen partial pressure. A mixture of water and isopropanol in a 1:2 *w/w* ratio was used as a solvent. Above the temperature of 120 °C the selectivity of the process decreased (87% at 149 °C and 79% at 175 °C), and the main by-product was a Tishchenko ester. While the hydrogen pressure in the range from 2.72 to 5.44 MPa did not affect the selectivity of NPG synthesis. The Ru/Al_2_O_3_ catalyst showed good reusability, and after three cycles no change in its activity was observed.

In another example [44], a Pt and Ru based catalyst was used for the HPA hydrogenation. In order to increase its activity and life span, the catalyst was enriched with vanadium. This catalyst can be deposited on the support, e.g., activated carbon, silica, alumina, zeolite, etc., which facilitates its separation from the reaction products. The process using this three-component catalyst on the activated carbon as a support runs with 100% selectivity and high HPA conversion (98–100%). The HPA hydrogenation methods using various catalysts are summarized in Table 1.

**Figure 6:**
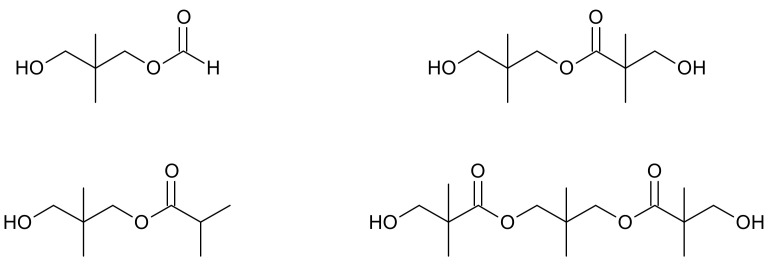
Examples of side products—esters, in the NPG synthesis [49].

In the case of the hydrogenation of crude hydroxypivaldehyde which contains side products such as esters (Figure 6), the problem is low conversion of these impurities to NPG. The patent [48] provides a solution to this problem by performing the catalytic hydrogenation of HPA to neopentyl glycol in two steps. The first stage is carried out at a temperature of 100 to 180 °C and a pressure of 1.5 to 4 MPa with the use of a copper-aluminum catalyst. In this stage, HPA is mainly hydrogenated. In the second stage, esters are hydrogenated at 200 to 250 °C and under a pressure of 3 to 5 MPa with the use of the copper/silicon catalyst. In another patent [49], esters that are separated after the NPG preparation process are hydrogenated in a separate reactor. The process is carried out at a temperature ranging from 140 to 220 °C. Copper chromite is used as a catalyst. Another method is described in the patent [50] and is based on the conversion of esters to NPG by hydrolysis during rectification with water. 

A patent by Aristech Chemical describes the method which allows the attainment of NPG with high efficiency and purity [45]. Neopentyl glycol is synthesized from isobutyraldehyde and formaldehyde or paraformaldehyde. The aldol product is mixed with a lower aliphatic alcohol to promote hydrogenation and allows the recovery of a high purity product by simple distillation. The developed method allows the avoidance of the need for additional processes to purify NPG from impurities such as saponification, neutralization, and extraction. The IBA, FA, and tertiary amine catalyst (triethylamine) are placed in a reactor and the mixture is stirred at 60–80 °C until most of the IBAL is consumed. The molar ratio of formaldehyde to isobutyraldehyde is from 0.5:1 to 2:1. Before the hydrogenation process, methanol is added as a solvent. The hydrogenation catalyst is copper chromite. The process is carried out at 160 °C and pressure of about 6.9 MPa.

### 2.3. Apparatus Improvement

An interesting equipment solution for the HPA hydrogenation reaction was presented by the Nan Ya Plastics Corporation [47]. The hydrogenation reactor was equipped with a device with a self-priming agitator. According to the invention, the self-aspirator is a special construction device which consists of a rotary hollow shaft with gas suction holes in the upper part and vent holes with a propeller in the lower part. This device is placed in a gas-tight reactor. A self-priming agitator ensures the effective gas circulation and gas-liquid contact. Therefore, there is no need to carry out the process under high pressure. The hydrogenation can be carried out at a temperature of 70 to 120 °C, a pressure of 0.6 to 12.4 MPa, and the reaction time should range from 1 to 6 h. The Raney nickel promoted with molybdenium has been proposed as a catalyst. The amount of catalyst varies from about 0.2 to 15 wt% of the hydroxypivaldehyde. Before the hydrogenation, the crude hydroxypivaldehyde stream can be directed to distillation in order to remove isobutyraldehyde and triethylamine, and diluted with an organic solvent, e.g., aliphatic alcohol. The conversion of HPA to NPG was 99.8% (3 MPa, 100 °C, 2 h, 500 rpm).

The authors of the patent [5] proposed an economical apparatus for carrying out the two-step synthesis of NPG by the aldol condensation of IBA and FA, as well as the subsequent hydrogenation of HPA. The apparatus consists of an aldol reactor which may be a jacketed reactor or a series of 2 to 5 continuous stirred-tank reactors. The reaction is carried out at the temperature of 70–95 °C in the presence of a tertiary amine as a catalyst. The resulting product is passed to an extractor, preferably a multi-stage extractor, where FA, trialkylamine salts, etc. are extracted with an alcohol that is immiscible with water, e.g., 2-ethylhexanol. The extract is then directed to the distillation column, where mainly the unreacted starting materials and catalyst are separated. Thereafter, the purified HPA stream is hydrogenated in the presence of a copper-based catalyst, e.g., CuO/BaO on a silica support, in a reactor, e.g., a fixed-bed reactor. Another apparatus is a divided-wall distillation column, which allows the attainment of NPG with a purity above 99.5%. 

## 3. Conclusions

Neopentyl glycol, due to its properties and wide application, is the subject of numerous studies aimed at improving the methods of its synthesis. This is evidenced, in particular, by the constantly growing number of patents. The research focuses mainly on the improvement of the catalysts of both the crossed aldol condensation and hydrogenation stage. Increasing the activity and selectivity of the catalysts for these reactions will allow the reduction of the consumption of raw materials and auxiliary reagents, in order to conduct the reaction under milder process conditions and to shorten the reaction time. In addition, research is underway to search for active and contamination-resistant heterogeneous catalysts that can be easily separated from the reaction mixture and used in continuous processes. The purification units for both the HPA intermediate and the final NPG product require an upgrade by improving the existing separation techniques or introducing new, more energy-efficient methods. Improvements in the apparatus, e.g., the reactor, are also sought. All of these activities are aimed at developing low-waste, material-and energy-saving techniques for the production of NPG that will be cost-effective on an industrial scale.

## Figures and Tables

**Table 1:** Comparison of NPG preparation methods by HPA hydrogenation.

Catalyst	Temperature, °C	Pressure, MPa	Solvent	HPA Conversion, %	Yield, %	Selectivity, %	Ref.
manganese oxide-promoted copper chromite	150–220	2.0–10.3	-	-	90.6	-	[30]
copper chromite	100–200	3.5–20.7	aliphatic alcohol (20–90 wt%) water (0–40%)	-	-	99 (purity)	[45]
copper chromite with barium and manganese as activators	80–140	2.0–18.0	aliphatic alcohol (20–90 wt%) water (15–25 wt%)	99.4–99.9	-	94–100	[31]
copper chromite with barium as activator	I stage: 120–160 II stage: 170–200	20.0–30.0	water 3–8 wt%	-	>99	-	[29]
copper chromite with barium and manganese as activators	125–180	0.03–0.12	water (15–25 wt%)	>99.8	-	97.3–98.6	[46]
copper, zinc, and zirconium	60–250	0.1–14.7	alcohol, ether or saturated hydrocarbons	99.7–100	-	97.6–99.0	[32]
copper chromite or ruthenium activated carbon (containing Cu, Co, Mn) copper chromite or ruthenium activated carbon (containing Cu, Co, Mn)	110–170	2–6	-	98.1	-	99.3	[3]
copper-based catalyst, e.g., CuO/BaO	100–250	1.0–25.0	NPG (35–70 wt%), alcohol (10–30 wt%) and water (10–30 wt%)	99.2–99.8	99.2–99.7	-	[28]
copper chromite, oxides of cobalt, manganese, nickel, Pt, Ru, W, Pd	50–200	0.1–20	alcohol, ether, ketone (1–70 wt%)	98.1	-	90.7	[27]
copper oxide and zinc oxide with aluminum as a promoter	110–180	0.1–3.45	alcohol	100	100	-	[33]
nickel-containing catalyst	<100		aliphatic alcohol or ether or their mixture (1–70 wt%) water (0–15 wt%)	91.0–93.2	-	98.5–99.7	[4]
molybdenum promoted Raney nickel	70–120	0.6–12.4	aliphatic alcohol (16–54 wt%)	99.8	-	--	[47]
nickel-based catalyst, e.g., Raney nickel	120–180	0.7–10.3	-	-	-	98 (purity)	[20]
Raney cobalt	60–220	2.0–15.0	linear or branched alcohol	>99.9	-	-	[43]
Ru/Al_2_O_3_	120	5.4	water and isopropanol (1:2 *w/w* ratio)	-	-	>99	[22]
						
metals from transition groups, such as Fe, Ru, Os, Co, Rh, Ir, Ni, Pd, Pt, Cu, and most often Ni and Cu, on a support, e.g., titanium oxides	50–180	1.0–25.0	water, cyclic or acyclic ethers, lower alcohols	99.9	-	97.9	[6]
Pt-Ru-W	60–150	0.1–4.9	water	98.0–100		100	[44]

## Data Availability

Data sharing is not applicable for this article.
